# Supplementary material for: A Micromechanical Study of Interactions of Cyanate Ester Monomer with Graphene or Boron Nitride Monolayer
Source: Materials (Basel). 2023 Dec 25;17(1):108. doi: 10.3390/ma17010108 (PMC10780284; doi:10.3390/ma17010108)
Supplement: Supplementary file 1 [file materials-17-00108-s001.zip › materials-2784596-supplementary.pdf]

Supporting Information

A Micromechanical Study of Interactions of Cyanate Ester Monomer with Graphene or BN Monolayer

Geeta Sachdeva[1*], Álvaro Lobato[2], Ravindra Pandey[1*,] and Gregory M. Odegard[3]

[1]*Department of Physics, Michigan Technological University, Houghton, MI 49931*

[2]*MALTA-Consolider Team and Departamento de Química Física y Analítica, Universidad de Oviedo, E-33006 Oviedo, Spain*

[3]*Department of Mechanical Engineering and Engineering Mechanics, Michigan Technological University, Houghton, MI 49931*

December 25, 2023

*Email: gsachdev@mtu.edu, pandey@mtu.edu

Table S1: Some of the representative structural parameters of the fluorinated and non-fluorinated cyanate ester monomers.

	AroCy-F10	*Primaset PT-30*
Binding energy/atom (eV)	-4.3	-4.1
Bond distance, C-C (Å)	1.40	1.40
C-H (Å)	1.09	1.09
C-O (Å)	1.41	1.30
C-N (Å)	1.17	1.17
C-F (Å)	1.35	-
Bond angle, C-C-C (°) (Benzene ring)	121.4	119.6

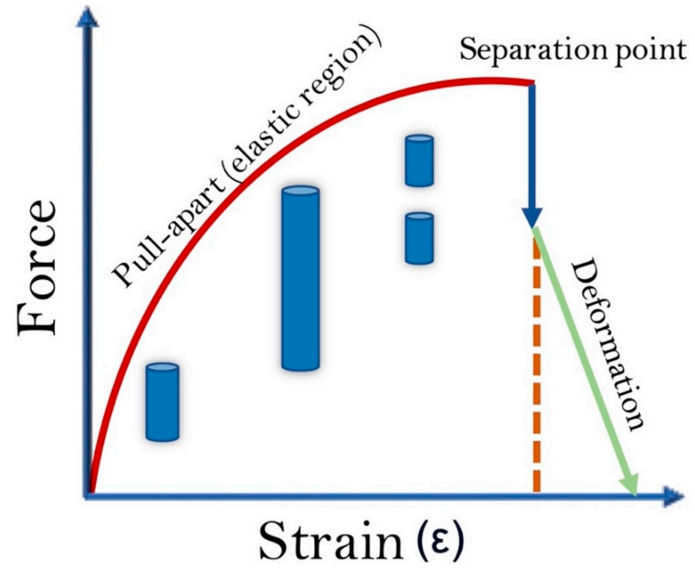

Figure S1: Schematic of force-strain relationship during pull-apart mechanism, where the monomer complex separates into its constituents at the point of maximum force. A solid geometry represents the monomer complex in the figure.

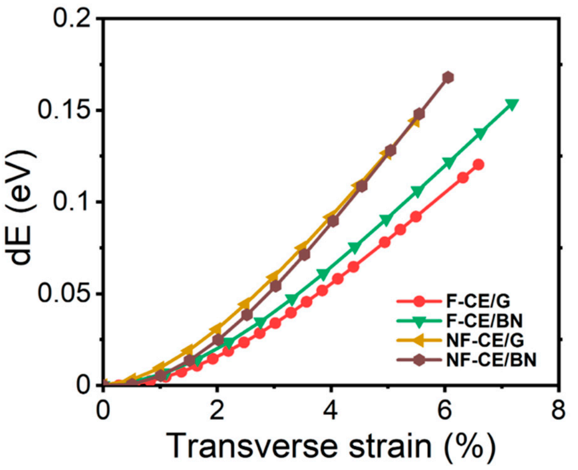

Figure S2: The calculated strain-energy relationship of the fluorinated (F-CE) and non-fluorinated cyanate ester (NF-CE) interacting with graphene (G) and BN monolayer. Zero is taken to be the equilibrium configuration, and dE is the energy with respect to the total energy of the equilibration configuration.

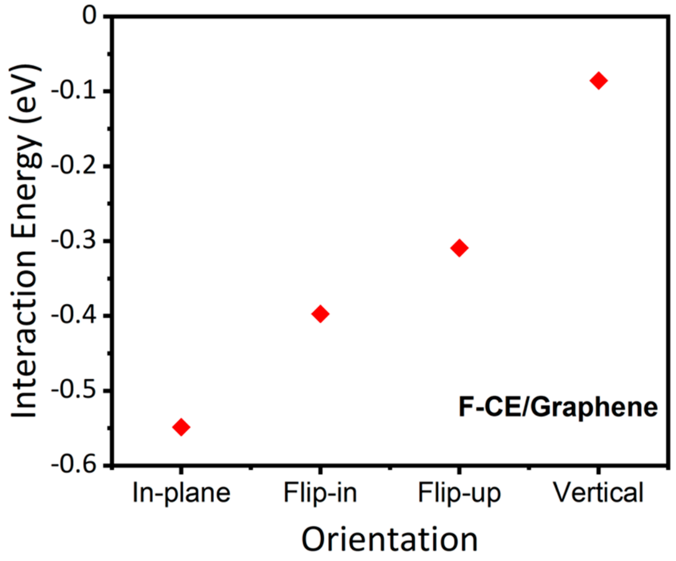

Figure S3: Orientation dependent interaction energy for flourinated cyanate ester monomer with graphene monolayer

Figure S4 presents the total electronic density of states (DOS) and density of states of individual components for both fluorinated and non-fluorinated cyanate ester with graphene monolayer between –10 and 4 eV, where the Fermi level is set at zero. It is important to note that the electronic energy distribution determines the probability that a given energy state will be occupied, but that probability is multiplied by the density of states function to account for the number of possible states at that energy level [1].

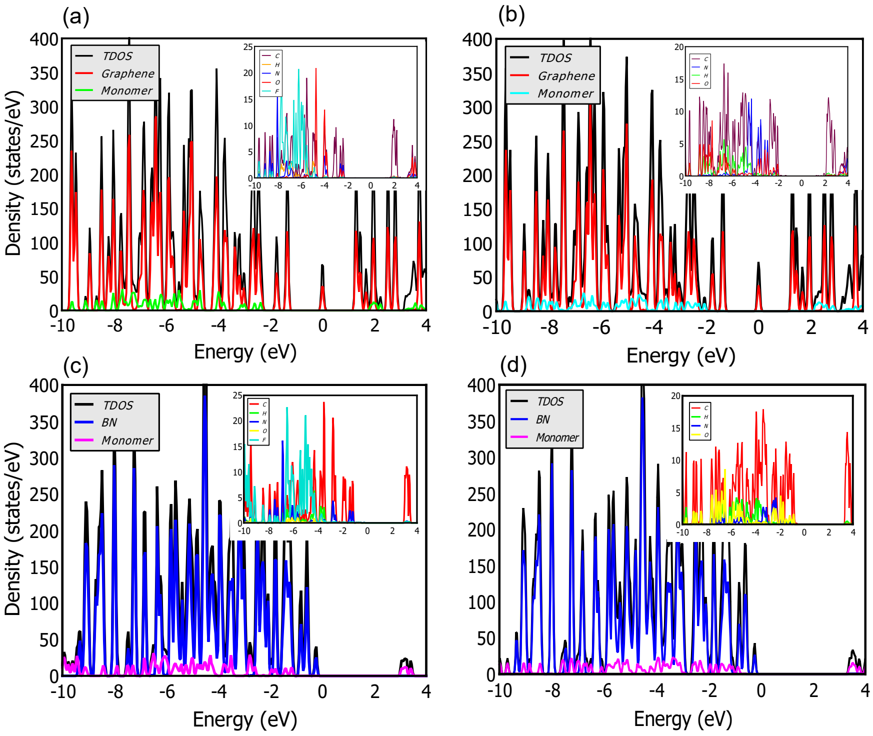

Figure S4: Calculated total density of states and density of states for monomers forming composites with graphene or BN monolayer: (a) fluorinated ester/graphene, (b) non-fluorinated ester/graphene, (c) fluorinated ester/BN, (d) non-fluorinated ester/BN. The inset shows DOS contributions from the corresponding monomers in the complexes.

Table S2: Calculated mechanical response in terms of Transverse strength, (out-of-plane) Separation point, Transverse stress, Stiffness, and fitting constant γ of the monomer complexes.

Complex		*Transverse strength* f_c *(nN)*	*(out-of-plane) Separation point* ε_c *(%)*	*Transverse stress (GPa)*	*Stiffness*	γ
Graphene	AroCy-F10 (fluorinated)	0.57	5.9	0.6	0.18	0.65
Graphene	Primaset PT-30 (non-fluorinated)	0.71	5.2	0.7	0.26	0.76
BN monolayer	AroCy-F10 (fluorinated)	0.63	6.4	0.7	0.20	0.54
BN monolayer	Primaset PT-30 (non-fluorinated)	0.80	5.8	0.8	0.25	0.63

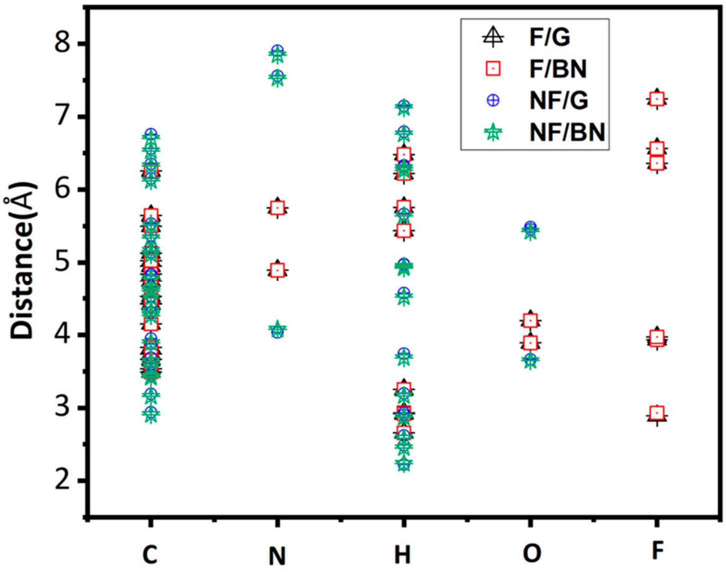

Figure S5: The distances between the atoms of the fluorinated and non-fluorinated ester monomer with nearest C atom of graphene and nearest B or N atom of BN monolayer.

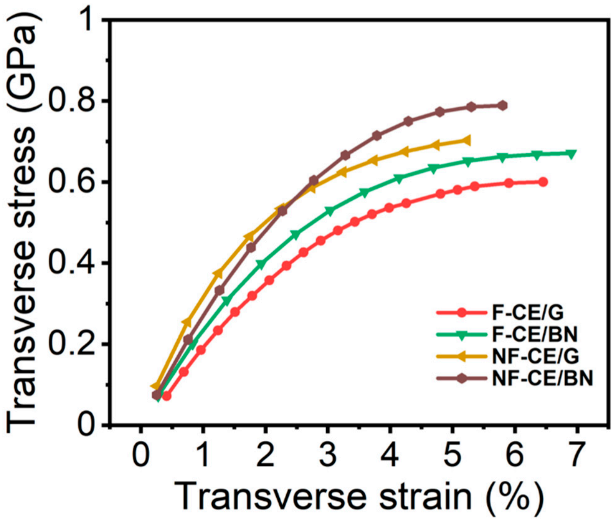

Figure S6: Calculated Transverse stress vs. Transverse strain curve for the fluorinated (F-CE) and non-fluorinated cyanate ester (NF-CE) forming complexes with graphene (or BN monolayer).

Figure S5 shows the distances between atoms of fluorinated and non-fluorinated ester monomers with the nearest C atom of graphene and the nearest B or N atom of the BN monolayer. The H atoms of the ester monomers serve as the nearest-neighbor atoms for the graphene (or BN) monolayer. The graph underscores the absence of N or O atom impact at the surface, revealing only a minor charge transfer at the interface, potentially attributed to H or C atoms of the monomer interacting with the surface. Notably, for the fluorinated monomer, interactions at the surface may be influenced by F atoms.

The computational outcomes in Figure S6 indicate a slightly greater transverse strain in the complexes formed between fluorinated cyanate ester and the BN monolayer than those with flCNT. Additionally, when considering fluorinated and non-fluorinated cyanate esters, the non-fluorinated ester paired with flCNT/BN demonstrates higher transverse stress than its fluorinated counterpart. Notably, the fluorinated ester is anticipated to possess a higher transverse strain than the non-fluorinated ester, contributing to improved interfacial load transfer. This phenomenon is attributed to the impact of fluorine atoms, introducing steric hindrance and interlocking effects in the fluorinated case. Consequently, as reported in previous reports, this impedes the separation of the fluorinated ester from the surface.

References

1. Kresse, G., A. Gil, and P. Sautet, *Significance of single-electron energies for the description of CO on Pt (111).* Physical Review B, 2003. **68**(7): p. 073401.
